# Supplementary material for: RHBDL4-triggered downregulation of COPII adaptor protein TMED7 suppresses TLR4-mediated inflammatory signaling
Source: Nat Commun. 2024 Mar 7;15:1528. doi: 10.1038/s41467-024-45615-2 (PMC10920636; doi:10.1038/s41467-024-45615-2)
Supplement: Supplementary file 1 — Supplementary Information [file 41467_2024_45615_MOESM1_ESM.pdf]

RHBDL4-triggered downregulation of COPII adaptor protein TMED7 suppresses TLR4-mediated inflammatory signaling

Julia D. Knopf[1,2,§], Susanne S. Steigleder[1,2,§], Friederike Korn[1,2], Nathalie Kühnle[1], Marina Badenes[3,#], Marina Tauber[2], Sebastian J. Theobald[4,5,6], Jan Rybniker[4,5,6], Colin Adrain[3,7], Marius K. Lemberg[1,2,*]

[1]Center for Molecular Biology of Heidelberg University (ZMBH), Heidelberg, Germany
[2]Center for Biochemistry and Cologne Excellence Cluster on Cellular Stress Responses in Aging-Associated Diseases (CECAD), Faculty of Medicine, University of Cologne; Cologne, Germany
[3]Instituto Gulbenkian de Ciência (IGC), Oeiras, Portugal
[4]Department I of Internal Medicine, Faculty of Medicine and University Hospital Cologne, University of Cologne, 50937 Cologne, Germany
[5]Center for Molecular Medicine Cologne (CMMC), University of Cologne, 50931 Cologne, Germany
[6]German Center for Infection Research (DZIF), Partner Site Bonn-Cologne, 50931 Cologne, Germany
[7]Patrick G Johnston Centre for Cancer Research, Queen's University Belfast, UK
[#]Present address: Faculty of Veterinary Medicine, Lusofona University and Faculty of Veterinary Nursing, Polytechnic Institute of Lusofonia, Lisbon, Portugal
[§]These authors contributed equally.
*Corresponding author: m.lemberg@uni-koeln.de

Supplementary Information

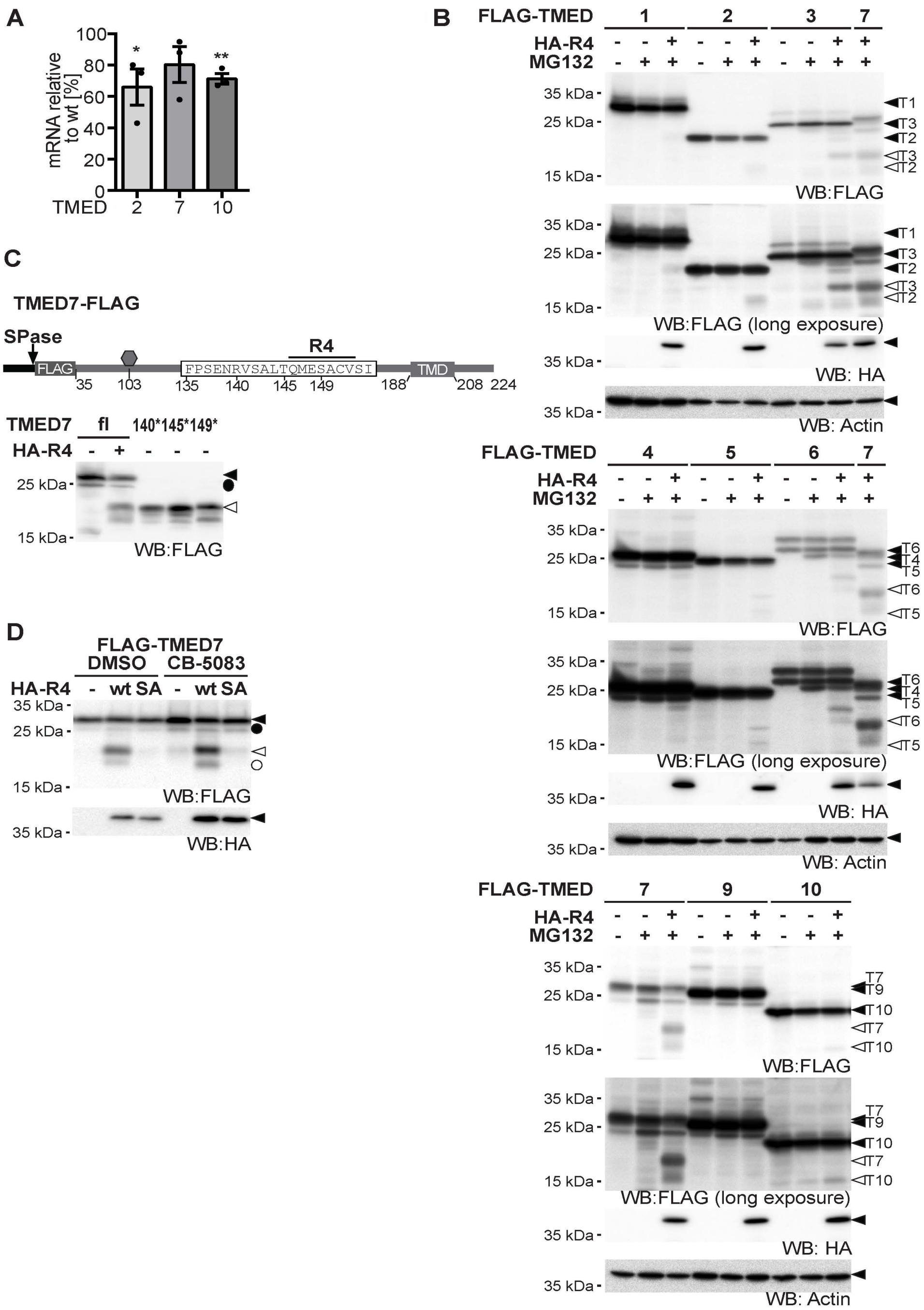

Supplementary Fig. 1. RHBDL4 cleaves certain TMED/p24 proteins to trigger their degradation along the ERAD pathway. Related to Fig. 1. (A) Transcriptional levels of TMED2, TMED7, and TMED10 in HEK293T R4 ko cells relative to wt cells revealed a modest downregulation. For TMED7 this did not reach significance (means ± SEM n=3, * $p < 0.05$, ** $p < 0.01$, Student's t-test) **(B)** HEK293T cells were co-transfected with the indicated FLAG-tagged TMED constructs (filled arrows) and either an empty vector (-) or HA-tagged RHBDL4 (HA-R4) wt. For TMED1, -2, -3, -4, -5, -6, -7, and -10, RHBDL4 generates N-terminal cleavage fragments (open arrows). Where indicated, proteasome inhibitor MG132 (2 µM) was added. For comparison, all western blots (WB) show the TMED7 cleavage assay.

Actin is used as a loading control (n = 3). **(C)** HEK293T cells were co-transfected with either FLAG-TMED7 and empty vector (-) or HA-R4. In addition, FLAG-TMED7 variants truncated at position 140 (140*), 145 (145*), and 149 (149*) were transfected as reference peptides for the RHBDL4-generated cleavage fragment. Cells were treated with MG132 (2 μM) (n = 2). **(D)** HEK293T cells were co-transfected with FLAG-TMED7 (filled arrows) and either an empty vector (-), HA-R4 wt or the catalytically inactive SA mutant. RHBDL4 generates N-terminal cleavage fragments (open arrow) that are degraded by the proteasome as shown by increased steady-state level upon CB-5083 treatment (1 μM) compared to vehicle control (DMSO) (n = 3). Source data are provided as a Source Data file.

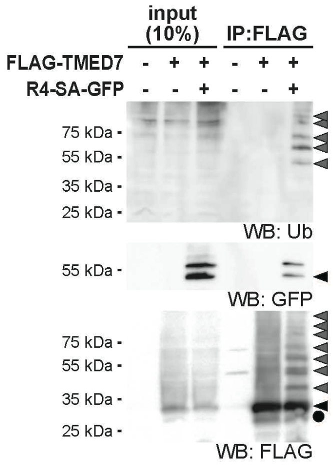

Supplementary Fig. 2. RHBDL4 interacts with ubiquitinated TMED7. Related to Fig. 2. HEK293T cells were transfected with FLAG-TMED7 and either empty vector (-) or GFP-tagged RHBDL4 SA (R4-SA-GFP) were lysed with Triton X-100 and subjected to FLAG-specific immunoprecipitation (IP). Western blot (WB) analysis reveals increased co-purification of ubiquitinated (Ub) FLAG-TMED7 species (grey arrows) upon transfection of R4-SA-GFP (n = 4). Source data are provided as a Source Data file.

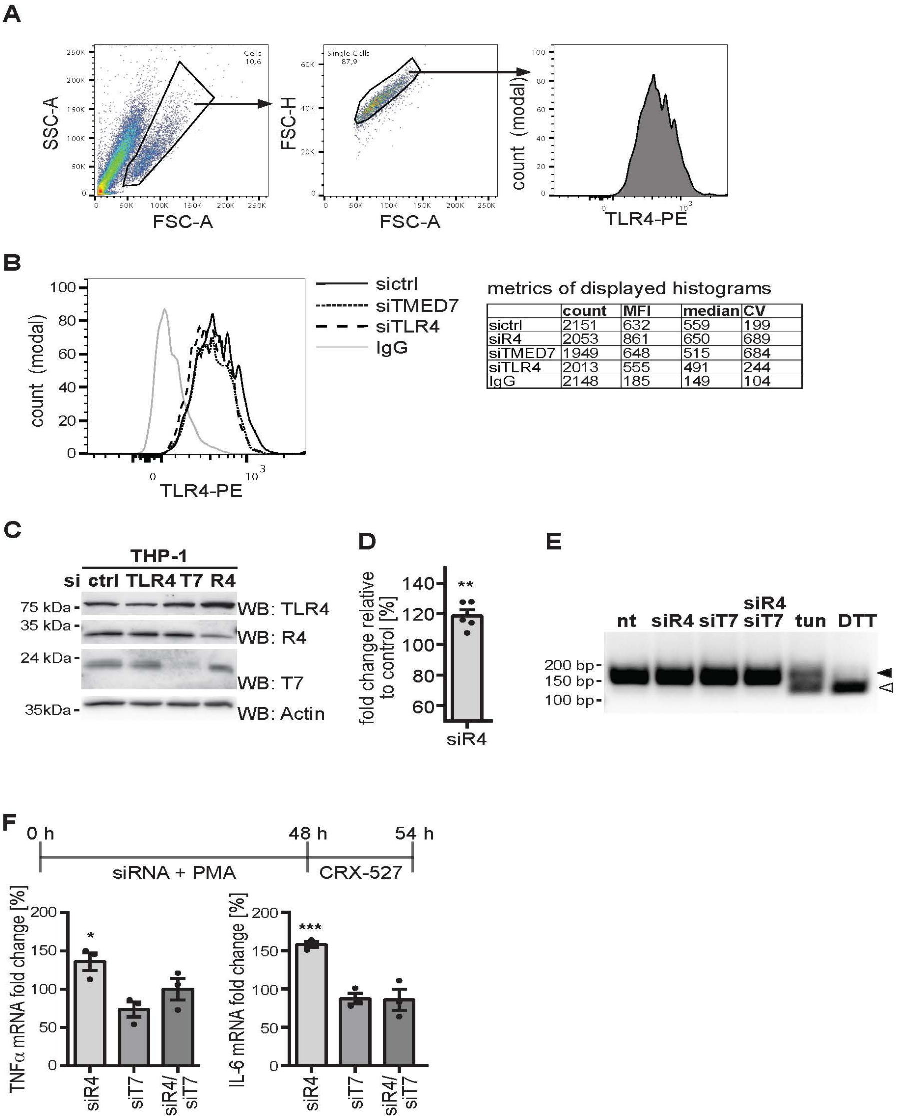

	count	MFI	median	CV
sictrl	2151	632	559	199
siR4	2053	861	650	689
siTMED7	1949	648	515	684
siTLR4	2013	555	491	244
IgG	2148	185	149	104

Supplementary Fig. 3. Loss of RHBDL4 in THP-1 cells leads to TMED7 stabilization and consequently to increased TLR4 cell surface level and signaling. Related to Fig. 3. **(A)** Gating strategy for data shown in Fig. 3B and Supplementary Fig. 3B. Differentiated THP-1 cells (high FSC, FSC-H) have been gated based on FSC and side scatter, doublets have been excluded and histograms showing PE staining of single cells have been reported. **(B)**TLR4 staining control for cell surface levels on cells treated with siRNA targeting TLR4 (siTLR4) and TMED7 (siTMED7) (n=2). Cells have been stained with anti-TLR4-PE and analyzed by flow cytometry. Grey curve represents PE-coupled isotypic control (IgG) and straight curve represents sictrl-treated cells as shown in Fig. 3B. **(C)** Western blot (WB) analysis to validate the knockdown of RHBDL4 (R4), TMED7, and TLR4 in the cells used for the flow cytometry shown in Fig. 3B and Supplementary Fig. 3A. Actin is used as a loading control. Equal protein loading was controlled on a separate WB (n = 2). **(D)** NF-κB activity in THP-1 cells was measured by a stably expressed secreted luciferase reporter. RHBDL4 knockdown (siR4) leads to a subtle but significant increase of LPS-induced (0.1 µg/ml) NF-κB activity relative to the LPS-treated control (means ± SEM n=5, ** $p < 0.01$, Student's t-

test). **(E)** No ER stress and UPR was observed in PMA-treated THP-1 cells upon knockdown of RHBDL4 and TMED7. THP-1 cells were transfected either with sictrl (nt), siR4, siT7 or a combination of the latter two (as in Fig. 2B) and UPR activation was assessed by the distribution of spliced (activated) and unspliced (inactive) XBP1 mRNA by RT-PCR and agarose gel electrophoresis. As positive control for UPR activation, THP-1 cells were treated with tunicamycin (tun, 2 µg/ml) and DTT (2 µM) for 2h (n = 3). **(F)** THP-1 cells were transfected either with sictrl, siR4, siT7 or a combination of the latter two and treated with CRX-527 (0.1 µg/ml) for 6 h. Normalized transcriptional levels of TNFα and IL-6 relative to CRX-527-treated control cells. Both TNFα and IL-6 expression increases upon knockdown of RHBDL4 (means ± SEM, n=3; * $p < 0.05$, *** $p < 0.001$, Student's t-test). Source data are provided as a Source Data file.

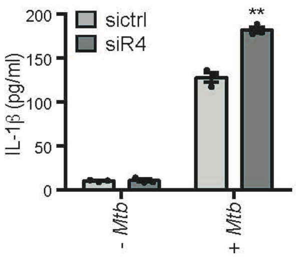

Supplementary Fig. 4. Pathophysiological consequence of RHBDL4 ablation. Related to Fig. 4. Amount of IL-1β determined by ELISA in the supernatant of THP-1 cells transfected with siRNA targeting RHBDL4 (siR4) or control siRNA (sictrl) upon infection with *M. tuberculosis* (+*Mtb*) for 24 h (means ± SEM, n=3; ** $p < 0.01$, Student's t-test). Source data are provided as a Source Data file.

A

```
Hs RHBDL4       MQRRSRGINTGLILLLSQIFHVGINNIPPVTLATL----ALNIWFFLNPQKPLYSSCLSV 56
Mm RHBDL4       MQRRTRGINTGLLLLLSQVFQIGINNIPPVTLATL----AVNVWFFLNPWKPLYHSCISV 56
Gg RHBDL4       MQRRQGRVNAGLLLLLYQISQVGLQNIPSVTLAVL----ILNVFFFLNPMRPLHEACISV 56
Xl RHBDL4       MQRRQRGINAGLVLLFAQLYQFGIHNIPPVTLAAL----GLNIYLFLNPLKPLLKVCISV 56
Dr RHBDL4       MRNRQRGTNLGLLLLASQVFQLGIDNIPPVTLATL----GLNVYLFLFPFKPLLETCLSV 56
At3g58460       MR-PNIVTEAGVQTRVGQW----WNAIPFLTSSVVVVCGVIYLICLLTGYDTFYEVCFLP 55
Os03g44830      MR-PNVITEAGISTRLNQW----WGSIPFITSGVILICGAIYLVCLLTGYDSFAEICFLP 55
                *:      : *:     *        ** :* ..:     : :  :*     :   *:

Hs RHBDL4       EKCYQQKDWQRLLLSPLHHADDWHLYFNMASMLWKGINLERRLGSRWFAYVITAFSVLTG 116
Mm RHBDL4       EKCYQQKDWQRLLLSPLHHGDDWHLYFNMVSMLWKGVKLERRLGSRWFAYVIATFSLLTG 116
Gg RHBDL4       KEAFYRQNWQRLLLSPVHHVDDWHLYYNMVSMLWKGIMLERKLGSVWFAYIIAVFSVLTG 116
Xl RHBDL4       REGYYGRDWQRFLLSPFHHADDWHLYFNMVSLLWKGAKLERRLGTGLFASIILVFSQLIG 116
Dr RHBDL4       REAYWYRDWSRLLLSPFHHVDDMHLYFNMALLLWKGIRLEKKLGGPWFAYLLSVFSLLTG 116
At3g58460       SAIISRFQVYRFYTAIIFHGSLLHVLFNMMALVPMGSELERIMGSVRLLYLTVLLATTNA 115
Os03g44830      SVVASRFQVYRFYTSVVFHGSLLHVLFNMLALVPLGTELERIMGSVRLLFLMFLLATTNA 115
                       :  *:  : ..* .  *: :**  ::  *  **: :*    :  :   ::   .

Hs RHBDL4       VVYLLLQFAVA--EFMDEPDFKRSCAVGFSGVLFALKVLNNHYCPGGFVNILGF-PVPNR 173
Mm RHBDL4       VVYLLLQFTVA--ELLNQPDFKRNCAVGFSGVLFALKVLSNHYCPGGFVNILGF-PVPNR 173
Gg RHBDL4       IVYVLLEFMLV--KILNDPSYEMNCGVGFSGVLFALKVLNNHYNPGRVSSVLGL-QISSK 173
Xl RHBDL4       VVYVILEYVLA--EFTGDPSFKMQCAVGFSGVLFALKVLNNHYHPGGSSNVFGI-LIPNK 173
Dr RHBDL4       FVYLLLETGLT--HMTEDSSYSLQCAVGFSGVLFGLKVVNNHYHPGGATNIMGL-PIANR 173
At3g58460       VLHLLIASLAGYNPFYQYDHLMNECAIGFSGILFSMIVIETSLSGVTSRSVFGLFNVPAK 175
Os03g44830      IFHLIIAFLAAYNPLYPLHFLVDECSIGFSGVIFSMIVIETSLSGVQTRSVFGLFNVPAN 175
                ..::::        :        .*.:****::*.: *:..        .::*:  :  .

Hs RHBDL4       FACWVELVAIHLFSPGTSFAGHLAGILVGLMYTQGPLKKIMEACAGGFSSSVGYPGRQYY 233
Mm RHBDL4       FACWAELVAIHFCTPGTSFAGHLAGILVGLMYTQGPLKKIMDTCAGIFISHAGPSGQQNH 233
Gg RHBDL4       YACWVELVAIHFIAPRTSFAGHLAGILVGLMYTMGPLKKIMKACAGGFSSFTDPDRPRND 233
Xl RHBDL4       YACWAELVAIHLLSPGTSFVGHLSGILVGLLYTQGPLKNILTASFS-----NGPSSAGRN 228
Dr RHBDL4       YACWVELVLIHIMNPGTSFVGHLSGILVGLLYTTGPLKRLMEICAGFVTSNGHYGGQQTY 233
At3g58460       LYPWILLIVFQLLMTNVSLLGHLCGILSGFSYSYGLFNFLMPGSS-FFTTIESASWMSSF 234
Os03g44830      WYAWILLVLFQFLASNVSLLGHLCGILSGFAYTYGLFNYLLPGPS-FYSSIEGLSVLSVC 234
                   *  *: :::    .*: ***.*** *: *: * :: ::

Hs RHBDL4       ------FNSS--GSSGYQDYYPHGR--PDHYEE---------------APRNYDTYTAGL 268
Mm RHBDL4       ------FNNA--GPSGYQNHYADGR--PVTYDA---------------TYRNYDVYTAGL 268
Gg RHBDL4       ------Y-------SGYSEYYR--Y--PDDQYR---------------TPNNYYDYTGGL 261
Xl RHBDL4       ------FNYS--GHSGYADYQYQQ----------------------HQSPRSYDFYTGGL 258
Dr RHBDL4       ------YNSS--GYSGYGMPYAPNTNYEQHYYGAPYRQQYNTNSAHTPTAPPQHPYTAGM 285
At3g58460       IRRPKFIMCTGGNPSSYIPTYSAQNTTSSGFSTGNAWRSL---SSW--LPQREAS----N 285
Os03g44830      VRRPGFILCTGGTTYGQLPTFSNTSTAPSALINANFLRNI---SSW--IPSRQTTTTQGN 289
                               .

Hs RHBDL4       SEEE----------------------QLERALQASLWDRG----NTRNSPPPY--GFHLS 300
Mm RHBDL4       SEEE----------------------QLERALRASIWDRG----NTRNGPMPY--GFRLP 300
Gg RHBDL4       TEEE----------------------QLERAVLNSLNERDFGGATYNNERRPY--GFWFP 297
Xl RHBDL4       NEDE----------------------QLQQAIRESLNERGFHISYCLPV------G---- 286
Dr RHBDL4       SEEQ----------------------QYEAALRASLNDRGGHTQ-ARSSHGP------FV 316
At3g58460       QSSEDSRFPGRGRTLSTARDPTAPAGETDPNLHARLLEDSSSPDRLSDATVNTVADSRQA 345
Os03g44830      TQEQDPRFPGRARTLSSAGTE-PTAREASANLHASLLDSTTPSDPLTSSQHPAANTVR-- 346
                 ..:                      : .  :   : :

Hs RHBDL4       P-E-----EMRRQRLHRFDSQ-------------------------------------   315
Mm RHBDL4       P-E-----EMRRQRLHRFDGQ-------------------------------------   315
Gg RHBDL4       P-EQHSEEEMRRQRLRRFWRQ-------------------------------------   317
Xl RHBDL4       ---HSCSVKMGIEGLNRMLLGHQQNNRL------------------------------   311
Dr RHBDL4       P-SNPSPEEIRWRRIQRFDS--------------------------------------   335
At3g58460       PIANAAVLPQSQGRVAASEEQIQKLVAMGFDRTQVEVALAAADDDLTVAVEILMSQQA   403
Os03g44830      -----ADATVAADQVDTFDEELKKLVGMGFEKTQAEVALAAADGDPNVAIEILMSQQG   399
                              :
```

B

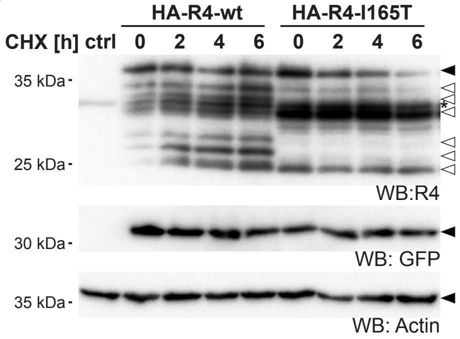

Supplementary Fig. 5. Kawasaki disease-associated mutation causes accelerated ERAD by a unique autocatalytic cleavage event in its first luminal loop. Related to Fig. 5. (A) Clustal Omega multi sequence alignment of RHBDL4 from *Homo sapiens (Hs), Mus musculus (Mm)*, *Gallus gallus (Gg)*, *Xenopus laevis (Xl)*, *Danio rerio (Dr)*, *Arabidopsis thaliana* (At), and *Oryza sativa (Os)*. Conserved hydrophobic residue affected by the Kawasaki mutation is highlighted in red; active site motifs (GFSG, H) are shown in bold. **(B)** Full-length form (filled arrow) of RHBDL4-IT (HA-R4-I165T) expressed in Hek293T cells is turned over faster compared to HA-tagged RHBDL4 wt (HA-R4-wt) when analyzed by cycloheximide (CHX) chase. Long exposure of the western blot reveals multiple degradation

intermediates (open arrows). Actin and co-transfected GFP are used as a loading and transfection controls, respectively and were controlled on a separate WB. Endogenous RHBDL4: asterisk. See Fig. 5C for quantification of HA-tagged full-length RHBDL4 (n = 5). Source data are provided as a Source Data file.

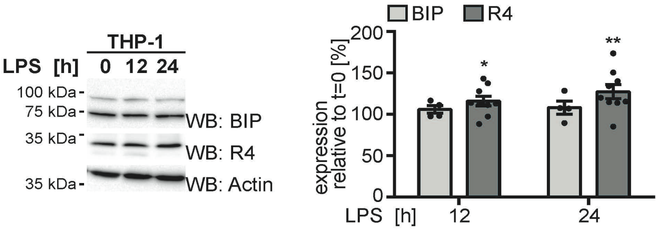

Supplementary Fig. 6. Negative feedback regulation: TLR4 stimulation upregulates RHBDL4 expression. Related to Fig. 6. RHBDL4 protein expression increases in THP-1 cells after treatment with LPS (1 µg/ml) for the indicated time points as assessed by western blot (WB) analysis. BiP expression was additionally monitored. Actin is used as a loading control. Right panel, quantification of RHBDL4 (n = 9) and BiP (n = 4) expression relative to untreated control (means ± SEM, * $p < 0.05$, ** $p < 0.01$, Student's t-test). Source data are provided as a Source Data file.

A

```
α
Hs_TMED04 ----------------------LYFHIGETEKRCFIEEIPDETMVIG-NYRTQMWDKQKEVFLPSTPGLGMHVEVKDPD--GKVVLSRQYGS
Hs_TMED09 ----------------------LYFHIGETEKKCFIEEIPDETMVIG-NYRTQLYDKQREEYQPATPGLGMFVEVKDPE--DKVILARQYGS
β
Hs_TMED02 ----------------------YFVSIDAHAEECFFERVTSGTKMG------LIFEVAEGG------FLDIDVEITGPD--NKGIYKGDRES
γ
Hs_TMED01 ------------AGPPPIQDGEFTFLLPAGRKQCFYQSAPANASLET------EYQVIGG------AGLDVDFTLESPQ--GVLLVSESRKA
Hs_TMED03 -----------------PCGAELTFELPDNAKQCFHEEVEQGVKFSL------DYQVITG------GHYDVDCYVEDPQ--GNTIYRETKKQ
Hs_TMED05 --------------FTPSLDSDFTFTLPAGQKECFYQPMPLKASLEI------EYQVLDG------AGLDIDFHLASPE--GKTLVFEQRKS
Hs_TMED06 QKTEPLSGSGDQPLFRGADRYDFAIMIPPGGTECFWQFAHQTGYFYFSYEVQRTVGMSHD--------RHVAATAHNPQGFLIDTSQGVRGQ
Hs_TMED07 --------------------SEITFELPDNAKQCFYEDIAQGTKCTL------EFQVITG------GHYDVDCRLEDPD--GKVLYKEMKKQ
δ
Hs_TMED10 ----------------------ISFHLPINSRKCLREEIHKDLLVTG----AYEISDQSGG----AGGLRSHLKITDSA--GHILYSKEDAT

α
Hs_TMED04 -EGRFTFTSHTPGDHQICLHSNSTRMALFAGGKLRVHLDIQVGEHANN-----------YPEIAAKDKLTELQLRARQLLDQVEQIQKEQDY
Hs_TMED09 -EGRFTFTSHTPGEHQICLHSNSTKFSLFAGGMLRVHLDIQVGEHAND-----------YAEIAAKDKLSELQLRVRQLVEQVEQIQKEQNY
β
Hs_TMED02 -SGKYTFAAHMDGTYKFCFSNR-MSTMTPKIVMFTIDIGEAPK----------------GQDMETEAHQNKLEEMINELAVAMTAVKHEQEY
γ
Hs_TMED01 -DGVHTVEPTEAGDYKLCFDNS-----FSTISEKLVFFELIFDSLQDD-EEVEGWAEAVEPEEMLDVKMEDIKESIETMRTRLERSIQMLTL
Hs_TMED03 -YDSFTYRAEVKGVYQFCFSNE-----FSTFSHKTVYFDFQVGDEPPIL----------PDMGNRVTALTQMESACVTIHEALKTVIDSQTH
Hs_TMED05 -DGVHTVET-EVGDYMFCFDNT-----FSTISEKVIFFELILDNMGEQAQEQEDWKKYITGTDILDMKLEDILESINSIKSRLSKSGHIQTL
Hs_TMED06 INFSTQETGFYQLCLSNQ---------HNHFGSVQVYLNFGVFYEGP-ETDHK--Q---KERKQLNDTLDAIEDGTQKVQNNIFHMWRYYNF
Hs_TMED07 -YDSFTFTASKNGTYKFCFSNE-----FSTFTHKTVYFDFQVGEDPPL-----------FPSENRVSALTQMESACVSIHEALKSVIDYQTH
δ
Hs_TMED10 -KGKFAFTTEDYDMFEVCFESK----GTGRIPDQLVILDMKHGVEAKN-----------YEEIAKVEKLKPLEVELRRLEDLSESIVNDFAY

α
Hs_TMED04 QR-YREERFRLTSESTNQRVLWWSIAQTVILILTGIWQMRHLKSFFEAKKLV-------
Hs_TMED09 QR-WREERFRQTSESTNQRVLWWSILQTLILVAIGVWQMRHLKSFFEAKKLV-------
β
Hs_TMED02 ME-VRERIHRAINDNTNSRVVLWSFFEALVLVAMTLGQIYYLKRFFEVRRVV-------
γ
Hs_TMED01 LR-AFEARDRNLQEGNLERVNFWSAVNVAVLLLVAVLQVCTLKRFFQDKRPVPT-----
Hs_TMED03 YR-LREAQDRARAEDLNSRVSYWSVGETIALFVVSFSQVLLLKSFFTEKRP-ISRAVHS
Hs_TMED05 LR-AFEARDRNIQESNFDRVNFWSMVNLVVMVVVSAIQVYMLKSLFEDKRKSRT-----
Hs_TMED06 AR-MRKMADFFLIQSNYNYVNWWSTAQSLVIILSGILQLYFLKRLFNVPTTTDTKKPRC
Hs_TMED07 FR-LREAQGRSRAEDLNTRVAYWSVGEALILLVVSIGQVFLLKSFFSDKRTTTTRVGS-
δ
Hs_TMED10 MK-KREEEMRDTNESTNTRVLYFSIFSMFCLIGLATWQVFYLRRFFKAKKLIE------
```

B

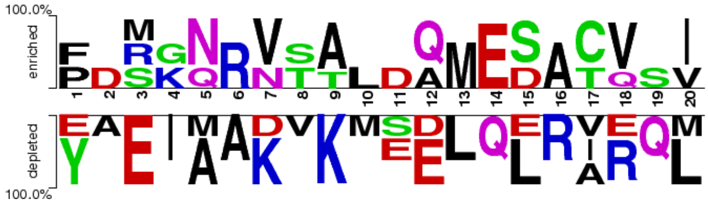

Supplementary Fig. 7. RHBDL4 cleaves a specific set of TMED proteins. (A) Multiple sequence alignment of the nine human TMED proteins was adapted from[60] and grouped according to their subfamilies. Green, GOLD domain; pink, 20 amino acids surrounding TMED7 cleavage site region and corresponding region in the TMED paralogs; turquoise, TM domain. **(B)** Two Sample Logo analysis[61] of the 20-amino acid residues around the TMED7 cleavage site region with the corresponding region in other TMED-paralogs from *Homo sapiens (Hs), Mus musculus (Mm), Rattus norvegicus (Rn), Pan troglodytes (Pt), and Macaca mulatta (Mm)*. TMED7, TMED3 and TMED6 were analyzed to identify enriched features while TMED9, TMED4 and TMED1 served for the analysis of depleted features.

Supplementary References

60. Strating JR, van Bakel NH, Leunissen JA, Martens GJ. A comprehensive overview of the vertebrate p24 family: identification of a novel tissue-specifically expressed member. *Mol Biol Evol* **26**, 1707-1714 (2009).

61. Vacic V, Iakoucheva LM, Radivojac P. Two Sample Logo: a graphical representation of the differences between two sets of sequence alignments. *Bioinformatics* **22**, 1536-1537 (2006).
