# Supplementary material for: RHBDL4-triggered downregulation of COPII adaptor protein TMED7 suppresses TLR4-mediated inflammatory signaling
Source: Nat Commun. 2024 Mar 7;15:1528. doi: 10.1038/s41467-024-45615-2 (PMC10920636; doi:10.1038/s41467-024-45615-2)
Supplement: Supplementary file 3 — Description of Additional Supplementary Files [file 41467_2024_45615_MOESM3_ESM.pdf]

Description of Additional Supplementary Files

Supplementary Data 1. Results from the proteome analysis comparing microsomes from SILAC-labelled Hek293T wt cells (light) with RHBDL4 knockout cells (heavy). Summary shows all ER-localized proteins that showed even a mild enrichment in knockout cells. Replicate 1 and 2 show a list of identified proteins.
