# Supplementary material for: RHBDL4-triggered downregulation of COPII adaptor protein TMED7 suppresses TLR4-mediated inflammatory signaling
Source: Nat Commun. 2024 Mar 7;15:1528. doi: 10.1038/s41467-024-45615-2 (PMC10920636; doi:10.1038/s41467-024-45615-2)
Supplement: Supplementary file 5 — Reporting Summary [file 41467_2024_45615_MOESM5_ESM.pdf]

nature portfolio

Corresponding author(s): MKL

Last updated by author(s): 15/01/24

# Reporting Summary

Nature Portfolio wishes to improve the reproducibility of the work that we publish. This form provides structure for consistency and transparency in reporting. For further information on Nature Portfolio policies, see our Editorial Policies and the Editorial Policy Checklist.

## Statistics

For all statistical analyses, confirm that the following items are present in the figure legend, table legend, main text, or Methods section.

| n/a | Confirmed | |
|---|---|---|
| ☐ | ☒ | The exact sample size (*n*) for each experimental group/condition, given as a discrete number and unit of measurement |
| ☐ | ☒ | A statement on whether measurements were taken from distinct samples or whether the same sample was measured repeatedly |
| ☐ | ☒ | The statistical test(s) used AND whether they are one- or two-sided<br>*Only common tests should be described solely by name; describe more complex techniques in the Methods section.* |
| ☒ | ☐ | A description of all covariates tested |
| ☒ | ☐ | A description of any assumptions or corrections, such as tests of normality and adjustment for multiple comparisons |
| ☐ | ☒ | A full description of the statistical parameters including central tendency (e.g. means) or other basic estimates (e.g. regression coefficient) AND variation (e.g. standard deviation) or associated estimates of uncertainty (e.g. confidence intervals) |
| ☐ | ☒ | For null hypothesis testing, the test statistic (e.g. *F*, *t*, *r*) with confidence intervals, effect sizes, degrees of freedom and *P* value noted<br>*Give P values as exact values whenever suitable.* |
| ☒ | ☐ | For Bayesian analysis, information on the choice of priors and Markov chain Monte Carlo settings |
| ☒ | ☐ | For hierarchical and complex designs, identification of the appropriate level for tests and full reporting of outcomes |
| ☒ | ☐ | Estimates of effect sizes (e.g. Cohen's *d*, Pearson's *r*), indicating how they were calculated |

*Our web collection on statistics for biologists contains articles on many of the points above.*

## Software and code

Policy information about availability of computer code

| | |
|---|---|
| Data collection | qPCR acquisition: Roche Light Cycler SW 1.51, Agarose gel imaging: BioRad Image Lab 6.1, Western blot acquisition: ImageQuant LAS 4000 or ImageQuant 800 2.0.0 analysis software, Flow Cytometry: BD FACSDiva v8.0.2, ELISA acquisition: Tecan i-control v1.10.4.0 or Hidex, Luminescence measurement: Tecan SPARKCONTROL V3.2 |
| Data analysis | Mass spectrometry data analysis: Perseus (1.6.14.0), Statistical analysis and data visualization: Graphpad PRISM version 6; Flow Cytometry: Flow Jo (v 10.2); Image analysis: FIJI Image J 2.0.0-rc-69/1.52p software (National Health Institute, MD, USA) , RHBDL4 Homology model: PyMOL Molecular Graphics System (v.2.2.0), qPCR/Luminescence/WB quantification: Microsoft Office 2016 Excel, Multiple sequence alignment: EMBL-EBI Clustal Omega (Analysis Tool Web Services from the EMBL-EBI. (2013) McWilliam H, Li W, Uludag M, Squizzato S, Park YM, Buso N, Cowley AP, Lopez R Nucleic acids research 2013 Jul;41(Web Server issue):W597-600 doi:10.1093/nar/gkt376 ) , Two Sample logo analysis: Two Sample logo ( Vacic V., Iakoucheva L.M., and Radivojac P. "Two Sample Logo: A Graphical Representation of the Differences between Two Sets of Sequence Alignments." Bioinformatics, 22(12): 1536-1537. (2006)) |

For manuscripts utilizing custom algorithms or software that are central to the research but not yet described in published literature, software must be made available to editors and reviewers. We strongly encourage code deposition in a community repository (e.g. GitHub). See the Nature Portfolio guidelines for submitting code & software for further information.

## Data

Policy information about availability of data

All manuscripts must include a data availability statement. This statement should provide the following information, where applicable:

- Accession codes, unique identifiers, or web links for publicly available datasets
- A description of any restrictions on data availability
- For clinical datasets or third party data, please ensure that the statement adheres to our policy

The authors declare that the data supporting the findings of this study are available within the paper and its supplementary information files. Source data are provided in the Source data file. The mass spectrometry proteomics data have been deposited to the ProteomeXchange Consortium via the PRIDE partner repository with the dataset identifier PXD045934 (https://www.ebi.ac.uk/pride/archive/projects/PXD045934).

## Research involving human participants, their data, or biological material

Policy information about studies with human participants or human data. See also policy information about sex, gender (identity/presentation), and sexual orientation and race, ethnicity and racism.

| | |
|---|---|
| Reporting on sex and gender | n/a |
| Reporting on race, ethnicity, or other socially relevant groupings | n/a |
| Population characteristics | n/a |
| Recruitment | n/a |
| Ethics oversight | n/a |

Note that full information on the approval of the study protocol must also be provided in the manuscript.

# Field-specific reporting

Please select the one below that is the best fit for your research. If you are not sure, read the appropriate sections before making your selection.

☒ Life sciences ☐ Behavioural & social sciences ☐ Ecological, evolutionary & environmental sciences

For a reference copy of the document with all sections, see nature.com/documents/nr-reporting-summary-flat.pdf

# Life sciences study design

All studies must disclose on these points even when the disclosure is negative.

| | |
|---|---|
| Sample size | n/a |
| Data exclusions | n/a |
| Replication | n/a |
| Randomization | n/a |
| Blinding | n/a |

# Reporting for specific materials, systems and methods

We require information from authors about some types of materials, experimental systems and methods used in many studies. Here, indicate whether each material, system or method listed is relevant to your study. If you are not sure if a list item applies to your research, read the appropriate section before selecting a response.

## Materials & experimental systems

| n/a | Involved in the study | |
|---|---|---|
| | x | Antibodies |
| | x | Eukaryotic cell lines |
| x | | Palaeontology and archaeology |
| | x | Animals and other organisms |
| x | | Clinical data |
| x | | Dual use research of concern |
| x | | Plants |

## Methods

| n/a | Involved in the study | |
|---|---|---|
| x | | ChIP-seq |
| | x | Flow cytometry |
| x | | MRI-based neuroimaging |

# Antibodies

| | |
|---|---|
| Antibodies used | beta-actin (clone AC-15, A1978, Sigma); anti-BiP (ab21685, Abcam); FLAG-HRP (clone: M2, A8592, Sigma), GFP (clones: 7.1 and 13.1, 11814460001, Roche), gp78 (clone 3D9, H00000267-M01, Novus Biologicals), anti-HA (3F10; 11867423001, Roche), anti-HA (clone 16B12, 901502, Biolegend, RHBDL4 (HPA013972, Sigma), TMED7 (gift from F. Wieland, Heidelberg University), anti-TMED2 (gift from F. Wieland), anti-TMED10 (gift from F. Wieland), anti-TLR4 (sc-293072, Santa Cruz Biotechnology), TLR4-PE (sc-13593, Santa Cruz Biotechnolog), ubiquitin (sc-8017, Santa Cruz Biotechnology) |
| Validation | mouse monoclonal anti-beta-actin (clone AC-15, A1978, Sigma) - validation by western blot in human cell lines by manufacturer (see https://www.sigmaaldrich.com/DE/de/product/sigma/a1978); rabbit anti-BiP (ab21685, Abcam) - validation by western blot in human cell lines by manufacturer (see https://www.abcam.com/products/primary-antibodies/grp78-bip-antibody-ab21685.html); mouse monoclonal anti-FLAG-HRP (clone M2, A8592, Sigma) - used in Lelek et al., 2015, Nature Communicatons, PMID 25744187 according to manufacturer; mouse monoclonal anti-GFP (clones: 7.1 and 13.1, 11814460001, Roche) - used in Thrun et al., 2021, Molecular Cell, PMID 33909987; mouse monoclonal anti-gp78 (clone 3D9, H00000267-M01, Novus Biologicals) - validated by siRNA and knockout experiments, data not shown, but can provided upon request; rat monoclonal anti-HA (clone 3F10, 11867431001, Roche) - used in Halbleib et al., 2017, Molecular Cell, , PMID 28689662; mouse monoclonal anti-HA (clone 16B12, 901502, Biolegend) - used in Kim et al., 2016, Nature Communications, PMID: 26757928; rabbit anti-RHBDL4 (HPA013972, Sigma) - validation by western blot in human cell line by manufacturer (see https://www.sigmaaldrich.com/DE/de/product/sigma/hpa013972) + knockdown validation by western blot is shown in Fig. S3C; rabbit anti-TMED7 (gift from F. Wieland, Heidelberg University) - knockdown validation by western blot is shown in Fig. S3C and also used in Jenne et al., 2001, Journal of Biological Chemistry, PMID 12237308); rabbit anti-TMED2 (gift from F. Wieland, Heidelberg University) - used in Jenne et al., 2001, Journal of Biological Chemistry, PMID 12237308); rabbit anti-TMED10 (gift from F. Wieland, Heidelberg University) - used in Jenne et al., 2001, Journal of Biological Chemistry, PMID 12237308; mouse monoclonal anti-TLR4 (clone: 25, sc-293072, Santa Cruz Biotechnology) - knockdown validation is shown in S3C; mouse monoclonal anti-TLR4-PE (clone: HTA125, sc-13593, Santa Cruz Biotechnology) - knockdown validation and comparison to isotype control is shown in S3B; mouse monoclonal anti-ubiquitin (clone P4D1, sc-8017, Santa Cruz Biotechnology) - validation by western blot in human cells with over-expressed ubiquitin by manufacturer (see https://www.scbt.com/p/ubiquitin-antibody-p4d1) and also used by Scinicariello et al., 2023, Elife, PMID 36961408. |

# Eukaryotic cell lines

Policy information about cell lines and Sex and Gender in Research

| | |
|---|---|
| Cell line source(s) | Hek293T - ATCC, catalog no. CRL-3216<br>Hek293T RHBDL4ko - based on Hek293T, described in Fleig et al., 2012 PMID 22795130<br>Hek293 T-REx Flp In (as parental cell line for all described Hek239 T-REx cell lines i.e. T-REx Flp In wt expressing an empty vector, T-REx Flp-In RHBDL4-ko expressing either an empty vector, HA-RHBDL4-wt, HA-RHBDL4-SA or HA-RHBDL4-IT) - Thermo Scientific, catalog no. R78007<br>THP-1 - gift from Georg Stoecklin, Mannheim University<br>THP-1 NF-kB Lucia - Invivogen, catalog no. thpl-nfkb |
| Authentication | None of the cell lines used were authenticated. |
| Mycoplasma contamination | All cell lines were regularly tested negative for mycoplasma contamination. |
| Commonly misidentified lines<br>(See ICLAC register) | n/a |

# Animals and other research organisms

Policy information about studies involving animals; ARRIVE guidelines recommended for reporting animal research, and Sex and Gender in Research

| | |
|---|---|
| Laboratory animals | All mice (Mus Musculus) used in this study were crossed in the C57BL/6J background. Age of the mice for LPS survival challenge was approximately 220 days. Mice for the LPS administration followed by blood collection were approximately 300 days of age. |
| Wild animals | The study did not involve wild animals. |

| | |
|---|---|
| Reporting on sex | Experiments were conducted in both male and female mice. However, for any experiment only mice from one sex were used: For the generation of bone marrow-derived macrophages female mice were used; for the LPS challenge, female mice were used; For the analysis of cytokine secretion and expression upon LPS administration, male mice were used. |
| Field-collected samples | The study did not involve field-collected samples. |
| Ethics oversight | Experiments with mice were performed in accordance with protocols approved by the Ethics Committee of the Instituto Gulbenkian de Ciência and the Portuguese National Entity Direção Geral de Alimentação e Veterinária (DGAV) and with the Portuguese (Decreto-Lei no.113/2013) and European (directive 2010/63/EU) legislation related to housing, husbandry, and animal welfare. |

Note that full information on the approval of the study protocol must also be provided in the manuscript.

# Plants

| | |
|---|---|
| Seed stocks | *Report on the source of all seed stocks or other plant material used. If applicable, state the seed stock centre and catalogue number. If plant specimens were collected from the field, describe the collection location, date and sampling procedures.* |
| Novel plant genotypes | *Describe the methods by which all novel plant genotypes were produced. This includes those generated by transgenic approaches, gene editing, chemical/radiation-based mutagenesis and hybridization. For transgenic lines, describe the transformation method, the number of independent lines analyzed and the generation upon which experiments were performed. For gene-edited lines, describe the editor used, the endogenous sequence targeted for editing, the targeting guide RNA sequence (if applicable) and how the editor was applied.* |
| Authentication | *Describe any authentication procedures for each seed stock used or novel genotype generated. Describe any experiments used to assess the effect of a mutation and, where applicable, how potential secondary effects (e.g. second site T-DNA insertions, mosiacism, off-target gene editing) were examined.* |

# Flow Cytometry

## Plots

Confirm that:

☒ The axis labels state the marker and fluorochrome used (e.g. CD4-FITC).

☒ The axis scales are clearly visible. Include numbers along axes only for bottom left plot of group (a 'group' is an analysis of identical markers).

☒ All plots are contour plots with outliers or pseudocolor plots.

☒ A numerical value for number of cells or percentage (with statistics) is provided.

## Methodology

| | |
|---|---|
| Sample preparation | For TLR4 cell surface analysis, 5x10^5 cells were seeded and transfected with siRNA, as described above. 48 h after transfection, the cells were washed with PBS and incubated in PBS on ice for 10 min. Cells were detached, spun down and resuspended in 50 µl PBS with 5% (v/v) FBS. 5 µl Fc Block (BD Biosciences) were added, and cells were incubated on ice for 25 min. Afterwards, the cells were stained with 10 µl phycoerythrin (PE)-coupled anti-TLR4 antibody or PE-coupled isotypic control for 20 min. Cells were washed once with PBS and resuspended in 300 µl PBS containing 5% (v/v) FBS. Cells were analyzed on a FACSCanto II (BD Biosciences). Forward light scatter, side light scatter, and fluorescence emission after excitation with 488 nm were acquired. The samples were analyzed using a FlowJo software, version 10.2. |
| Instrument | FACSCanto II (BD Biosciences) |
| Software | FlowJo software, version 10.2 |
| Cell population abundance | 5x10^5 cells |
| Gating strategy | Differentiated THP-1 cells (high FSC) have been gated based on FSC and SSC; doublets have been excluded and histograms showing PE staining of single cells have been reported |

☒ Tick this box to confirm that a figure exemplifying the gating strategy is provided in the Supplementary Information.
